# Agreement With Conjoined NPs Reflects Language Experience

**DOI:** 10.3389/fpsyg.2018.00489

**Published:** 2018-04-19

**Authors:** Heidi Lorimor, Nora C. Adams, Erica L. Middleton

**Affiliations:** ^1^Linguistics Lab, Department of Languages, Cultures and Linguistics, Bucknell University, Lewisburg, PA, United States; ^2^Department of Hearing and Speech Sciences, Vanderbilt University, Nashville, TN, United States; ^3^Language and Learning Lab, Moss Rehabilitation Research Institute, Elkins Park, PA, United States

**Keywords:** statistical learning, conjunctions, number agreement, psycholinguistics, subject–verb agreement, language production, notional number

## Abstract

An important question within psycholinguistic research is whether grammatical features, such as number values on nouns, are probabilistic or discrete. Similarly, researchers have debated whether grammatical specifications are only set for individual lexical items, or whether certain types of noun phrases (NPs) also obtain number valuations at the phrasal level. Through a corpus analysis and an oral production task, we show that conjoined NPs can take both singular and plural verb agreement and that notional number (i.e., the numerosity of the referent of the subject noun phrase) plays an important role in agreement with conjoined NPs. In two written production tasks, we show that participants who are exposed to plural (versus singular or unmarked) agreement with conjoined NPs in a biasing story are more likely to produce plural agreement with conjoined NPs on a subsequent production task. This suggests that, in addition to their sensitivity to notional information, conjoined NPs have probabilistic grammatical specifications that reflect their distributional properties in language. These results provide important evidence that grammatical number reflects language experience, and that this language experience impacts agreement at the phrasal level, and not just the lexical level.

## Introduction

An important question in language research concerns the nature of grammatical representations. Traditional linguistic analysis of subject–verb number agreement treats grammatical number specifications as discrete, an assumption explicitly adopted in some prominent models of lexical access (Levelt, 1989; Levelt et al., 1999) and agreement production (Eberhard et al., 2005). By this view, nouns bear discrete values (e.g., singular, plural, and dual), which speakers use to form grammatical agreement with the verb. Alternatively, in probabilistic models of language production, such as the Constraint Satisfaction model (Haskell and MacDonald, 2003), instead of bearing discrete number representations, nouns accumulate evidence for singular or plural verb agreement. In a system like this, a noun’s probabilistic number value is based on which verbs have previously co-occurred with that noun, and some nouns (even when grammatically singular) accumulate evidence for both singular and plural agreement because they co-occur with both singular and plural verbs.

A related question is whether grammatical number information, whether discrete or probabilistic, is carried only by individual nouns, or whether certain constructions, such as complex noun phrases (NPs), also carry grammatical information. In the Marking and Morphing model (Eberhard et al., 2005), no number information is stored at the phrasal level; instead, grammatical specifications are located on nouns and determiners, and through a process like feature percolation, the NP gains a grammatical number value. This value is then combined with notional number information “marked” directly from the message on the NP, and the combination of grammatical and notional information determines the form of the verb. Therefore, while there is a summed value that is obtained for the entire NP, the grammatical number values come from nouns and their determiners, and not from the NP construction itself. Alternatively, in a Constraint Satisfaction framework (Haskell and MacDonald, 2003), certain types of NPs might also gain evidence for singular or plural agreement, in addition to the evidence accumulated by the individual nouns within the NP. Both of these questions: whether grammatical number information is probabilistic or discrete, and whether grammatical number information can be a property of phrases as well as lexical agreement controllers, are important because they speak both to the nature of grammatical knowledge (Lau et al., 2017) and how it is learned.

Empirical studies of subject–verb agreement have shown that verbs do not always agree with the grammatical values of subject head nouns and that agreement is impacted by the type of head noun and by the presence of “attractor” nouns in the sentence. For example, verbs may sometimes take plural agreement in the presence of a singular head noun when intervening plural nouns are in the subject NP (Bock and Cutting, 1992). When the subject head noun is a collective noun, such as *group* or *class*, agreement is variable, and plural agreement is common with NPs like *the class of children* or *the group of tourists* (Haskell and MacDonald, 2003).

Notional number has been identified as a likely factor that accounts for some of the variability in subject–verb agreement. For example, collective NPs such as *the gang on the motorcycles* that afford a more notionally plural interpretation are associated with increased plural verb agreement (Humphreys and Bock, 2005), compared to NPs with less notionally plural interpretations, like *the gang near the motorcycles*. Other observations consistent with an influence of notional number on agreement involve conjoined NPs, like *drinking and driving*, which may be associated with singular agreement when it refers to the act of driving while intoxicated, but if the drinking and driving are done at separate times, then plural agreement may be expected (Morgan, 1972, 1984).

Prominent models of subject–verb agreement differ in terms of how they account for the variability in subject–verb agreement. According to the Constraint Satisfaction model of subject–verb number agreement (Haskell and MacDonald, 2003), the fact that nouns like *group* or *class* frequently occur with both singular and plural verbs means that these nouns accumulate evidence for both singular and plural agreement, giving them probabilistic number specifications. On the other hand, Marking and Morphing (Eberhard et al., 2005), which treats grammatical number as discrete, does not incorporate probabilistic number specifications at the level of the individual speaker. However, in Marking and Morphing, different speakers can have different discrete grammatical number specifications, such that *group* might be plural for one speaker, and singular for another (Bock et al., 2006). This allows the Marking and Morphing model to maintain discrete number valuations for each noun or for a class of nouns, while still accounting for the variability in agreement with collective nouns at the population level (Bock et al., 2006).

Another major difference between the Constraint Satisfaction model (Haskell and MacDonald, 2003) and Marking and Morphing (Eberhard et al., 2005) has to do with whether there are number specifications at the phrasal level, or whether number specifications are only available at the lexical level. While Marking and Morphing (Eberhard et al., 2005) does not allow for any additional grammatical specifications at the phrasal level, in the Constraint Satisfaction model, phrasal structures can accumulate evidence for singular or plural agreement, just as lexical items can. Haskell et al. (2010) provided evidence for phrasal level specifications through an implicit learning task in which groups read stories containing complex NPs with collective head nouns and either singular, plural, or neutral verb agreement. One group of participants read stories in which the complex NPs took plural verb agreement, like *A trio of famous violinists were now scheduled for opening night.* In a subsequent production task (Experiment 1), these participants produced more plural verb agreement with a new set of collective NPs than other groups of participants, who had been exposed to singular or neutral prime sentences (*A trio of famous violinists was…/A trio of famous violinists had now been…*). In Experiment 2, Haskell et al. (2010) showed that those who received plural verbs with collective NPs were more likely to produce plural agreement on non-collective NPs that had singular head nouns and plural local nouns (*The stamp on the tickets to the VIP boxes…*). Haskell et al. (2010) interpreted these findings as evidence of implicit learning of grammatical number at the phrasal level, arguing that language users keep track of the statistical distribution of verb agreement associated with complex NPs and that they adjust their agreement behavior to reflect the distributional patterns in the input, even across phrases that have different types of head nouns.

Conjoined NPs are an interesting test case for exploring these issues because there is no clear single lexical controller. Prima facie, conjoined NPs tend to refer to two things. Such notional plurality might dictate that conjoined NPs always take plural agreement except in rare cases when the referent is a single thing (e.g., the misdemeanor interpretation of “drinking and driving” above). However, counter to this expectation, Brehm and Bock (2017) observed statistically significant variability in agreement with conjoined NPs in a task manipulating concreteness of the constituent nouns and the grammatical number (singular versus plural) of the second noun. When the second noun was singular compared to plural, Brehm and Bock (2017) found increased abstractness was associated with increased singular agreement. An interpretation of this is that increased concreteness of the participating nouns was associated with enhanced individuation of their corresponding referents (and thus, notional plurality), suggestive of a relationship between notional number and agreement variability with conjoined NPs.

To build on this work, we experimentally elicited agreement with conjoined NPs and assessed conjoined NP agreement in a corpus analysis, which is important for determining whether conjoined NP agreement variability that may be observed in a laboratory task is also evident in naturalistic language use. Furthermore, in contrast to Brehm and Bock (2017) where concreteness of the constituent nouns was manipulated as a proxy for variations in notional number, we leveraged normative ratings of the experimental materials to directly assess the impact of notional number on conjoined NP agreement. Lastly, we evaluated whether distributional information can shift agreement with conjoined NPs to assess the role of implicit learning as a factor that—along with notional number—contributes to agreement variability with conjoined NPs.

We first report a corpus analysis aimed at formally measuring the variability of agreement among a large, unselected sample of conjoined NP tokens from an internet-based corpus search. To foreshadow the results, we found striking variability, showing that conjoined NPs frequently co-occurred with singular verbs in English, i.e., singular agreement should not be treated as a “special” case. Within this corpus analysis, we explored additional factors that contributed to such variability, such as constituent grammatical number and conjoined noun type. Then, in light of strong influences of constituent grammatical number on conjoined NP agreement, in an oral completion task focusing on conjoined singular nouns only, we formally assessed the role of notional information in agreement with conjoined NPs while also accounting for influences from conjoined noun type.

In the remaining two experiments, which involved written production tasks with a learning component based on Haskell et al. (2010), we evaluated whether probabilistic grammatical representations play a role in agreement with conjoined NPs. If speakers adjust their agreement patterns with conjoined NPs based on experience, this would provide evidence for probabilistic number representations at the phrasal level, as predicted by the Constraint Satisfaction model (Haskell and MacDonald, 2003).

## Corpus Analysis

In order to gain an overview of how agreement with conjoined NPs typically works in English, we conducted a corpus analysis on a large, unselected sample of conjoined NP tokens. To the extent that agreement was not uniformly plural, we explored additional aspects afforded by the data that may contribute to such variability such as constituent grammatical number. In a further analysis where we held constituent grammatical number constant, we considered whether agreement patterns would vary by conjoined noun type, which can serve as a proxy for notional number, as conjoined mass nouns *(the heat and humidity*) are more likely to be notionally singular than conjoined animates (*the dog and cat*) or other concrete nouns (Brehm and Bock, 2017).

### Method

Three thousand, four hundred and seventeen English sentences with conjoined noun phrase subjects were retrieved from the World Wide Web using the Linguist’s Search Engine (Resnik and Elkiss, 2005). All sentences were hand-screened for grammatical structure and language context. Three main types of sentences were excluded. First, sentences were excluded if the automatic parser in the Linguist’s Search Engine had misclassified the sentence, most often when the conjoined NP was embedded within a prepositional phrase and was therefore not the subject of the sentence. Second, items were excluded because of international internet domain extensions or non-native-like errors elsewhere in the sentence. The non-native-like errors were judged subjectively by the coder and did not include errors in subject–verb agreement. Instead, these errors comprised misspellings, misuse of articles, non-canonical word order, or other blatant syntactic violations. Third, sentences were excluded if they included elements that might influence subject–verb agreement yet were beyond the scope of this investigation, including proper names (e.g., *Pride and Prejudice*), academic papers (e.g., *Chomsky and Halle*), and lists containing more than two nouns (e.g., *milk, eggs, and cheese*).

### Results

Overall, of the 3417 sentences that were extracted from the World Wide Web, 1357 were considered “valid” according to the exclusionary principles discussed above. Of these 1357 sentences, 621 had verbs that were unmarked for number, leaving 736 sentences with conjoined NP subjects and number-marked verbs. Out of these 736 sentences, 203 (28%) of the sentences had singular verbs, and 533 (72%) had plural verbs. Sentences were then coded for whether each of the conjoined nouns was singular or plural and for the types of nouns in the conjoined NP, as will be detailed below, to better understand what factors predicted whether agreement with a particular conjoined NP would be singular or plural.

#### Effect of Noun Number

In order to determine the role of constituent grammatical number on agreement with conjoined NPs, we investigated whether the presence of plural markings on one or both of the individual nouns changed the rate of plural agreement using all 736 conjoined NPs from the corpus that had a number-marked verb. The number and percentage of singular and plural verbs for each of these noun number combinations is provided in **Table 1**.

**Table 1:** Distribution of responses by noun number and percentage of plural versus singular verbs in corpus analysis.

Noun number in conjoined noun phrases (NPs)	Singular verbs (% singular)	Plural verbs (% plural)
Singular–Singular (*the dog and cat*)	193 (40%)	290 (60%)
Singular–Plural (*the dog and cats*)	1 (1%)	90 (99%)
Plural–Singular (*the dogs and cat*)	8 (16%)	43 (84%)
Plural–Plural (*the dogs and cats*)	1 (1%)	110 (99%)

A Chi-square test of independence showed that there was a significant effect of noun number on agreement, χ^2^(3, *N* = 736) = 112.2, *p* < 0.001. This analysis showed that noun number and verb number were not independent. Instead, 40% of sentences had singular verb agreement when both of the nouns were singular, compared to 1% singular verb agreement when both of the nouns were plural. It is interesting to note that linear proximity of plural nouns played a role in subject–verb agreement in the corpus analysis; whenever the closest noun to the verb was plural, there was nearly universal plural agreement.

#### Conjunct Type

In the analysis of noun number, the patterns of agreement with conjoined NPs were influenced by the number specifications on the individual nouns in the conjoined NPs. To determine whether noun type affects verb agreement, the nouns in each conjoined NP were classified according to three types: animate count/collectives, inanimate count/collectives, and mass nouns.

In order to isolate the effect of noun type, we conducted a Chi-square test of independence, using only the conjoined NPs in which both conjoined nouns were singular and were of the same type (443 sentences). **Table 2** lists the number and percentage of singular and plural verbs with each conjunct type when both of the nouns in the conjoined NP were singular.

**Table 2:** Distribution of responses by noun type and percentage of plural versus singular verbs in corpus analysis, when both nouns were singular and of the same type.

Conjunct type	Singular verbs (% singular)	Plural verbs (% plural)
Animate count and collective	0 (0%)	96 (100%)
Inanimate count and collective	43 (38%)	71 (62%)
Mass	135 (58%)	98 (42%)

A Chi-square test of independence showed that conjunct type and verb number were not independent, χ^2^(2, *N* = 443) = 92.6, *p* < 0.001. When the conjoined nouns were inanimate, both singular and plural verbs were frequently produced, and singular agreement was most common among conjoined mass nouns in the corpus sample. However, no singular verbs were found in the corpus sample when both of the conjoined nouns were animate.

### Discussion

The corpus data provide naturalistic evidence that agreement with conjoined NPs in English is variable. Within the whole corpus, conjoined NPs agreed with singular verbs in 28% of the sentences. Noun number also played an important role, as conjoined NPs that included two singular nouns exhibited more singular agreement than conjoined NPs that included plural nouns. An additional analysis showed an effect of noun type as singular agreement was more common when the conjuncts were inanimate or mass nouns, compared to animate nouns.

In terms of the effect of noun number, the presence of a plural noun in any position made singular verb agreement less likely, consistent with Brehm and Bock (2017); numerically, this effect was larger when the noun closest to the verb was plural. The tendency of verbs to agree with the closest plural noun in our corpus data has been experimentally replicated by Keung and Staub (2016), and is consistent with a phenomenon known as “single conjunct agreement” (Aoun et al., 1994; Bhatt and Walkow, 2013; Marušič et al., 2015), in which some languages have the option of agreeing with one of the conjoined nouns instead of with the conjunction as a whole. While English is not generally seen as having the grammatical option of single conjunct agreement, and single conjunct agreement is most commonly found cross-linguistically with postverbal, rather than preverbal, subjects (Willer Gold et al., 2017), it is possible that the phenomenon of single conjunct agreement may also arise through processing mechanisms. Evidence for this comes from the fact that Keung and Staub (2016) found an “illusion of grammaticality” in an eye-tracking study, where the participants showed faster overall reading time when a singular verb followed a singular noun (^∗^*the keys and the lock is rusty*), compared to when both conjoined nouns were plural (*^∗^the keys and the lock****s*** *is rusty*). However, the role of linear order in agreement is not the primary focus of this paper.

Regarding the effect of noun type, mass nouns tend to refer to non-individuated entities (Middleton et al., 2004) which, along with inanimacy, may promote a notionally singular valuation of the conjoined NP referent. Thus, the effect of noun type in the corpus study provides indirect evidence for an effect of notional number on agreement variability in conjoined NPs. In Experiment 1, we directly investigated the role of notional number on agreement with conjoined NPs while accounting for noun type effects. In light of strong influences of constituent grammatical number in the corpus analysis, Experiment 1 employed an oral production task that included only conjoined singular nouns to verify that notional number accounts for variability in agreement when grammatical number is controlled.

## Oral Sentence Completion Task (Experiment 1)

In order to empirically evaluate the role of notional information in agreement with conjoined NPs in English, we performed an oral sentence completion task using conjoined NPs with singular nouns. We created sentence preambles to comprise a range of notional number valuations that included count nouns (e.g., *the violin and viola*), collective nouns (e.g., *the playground and arcade*), and mass nouns (e.g., *the dust and mold*) as the constituent nouns of the conjoined NPs. As all of the conjoined animate NPs in the corpus analysis demonstrated plural agreement, we included only inanimate nouns in the oral production task to provide maximum opportunity to observe singular agreement.

### Method

#### Participants

The participants were 66 undergraduates at the University of Illinois Urbana-Champaign who received a small payment or partial credit toward an introductory psychology course requirement. Two participants were excluded because they produced fewer than five scorable items. All participants were native speakers of American English. This study was carried out in accordance with the recommendations of the Office for the Protection of Research Subjects at the University of Illinois Urbana-Champaign. All participants gave written informed consent in accordance with the Declaration of Helsinki. The protocol was approved by the Institutional Review Board at the University of Illinois Urbana-Champaign.

#### Materials

The experimental preambles were conjoined NPs consisting of one definite determiner, two singular inanimate nouns, and the conjunction *and* (i.e., *The* NP *and* NP). We chose to use a single determiner rather than two determiners (i.e., *The NP and the NP*) to maximize our sensitivity to notional effects, as we were concerned that the second determiner might increase the individuation of items inside the conjoined NP (cf. Brehm and Bock, 2017). Out of the 80 experimental preambles, there were 16 conjoined NPs with collective nouns, 32 conjoined NPs with count nouns, and 32 conjoined NPs with mass nouns. Collective nouns were pluralizable and were judged, *a priori*, to be collections of individuals. Count nouns were also pluralizable but referred to individual items, in which component parts were less prominent. Mass nouns were categorized based on their inability to take a plural marking without changing the dominant semantic reading. The full set of preambles is provided in the Supplementary Materials. There were also 68 filler preambles consisting of a variety of NPs (e.g., singular and plural nouns, mass nouns, complex NPs, and conjoined NPs involving at least one plural noun).

All of the experimental and filler preambles were compiled into 16 lists and were recorded by a female native speaker of English. Each list contained four items with collective NPs, eight items with count NPs, and eight items with mass NPs. This generated four lists, and one-quarter of the experimental preambles appeared on each list. An additional four lists were generated by flipping the order of each conjunction (e.g., *the milk and cheese* →*the cheese and milk*), to control for idiosyncratic effects of noun ordering (Benor and Levy, 2006). Each list began with the same 12 fillers, and the remaining 56 fillers were interspersed among the experimental items pseudorandomly, with all the experimental items separated by at least two fillers, and no more than two preambles from the same experimental conditions ever occupying neighboring slots for experimental items in the lists. The remaining eight lists (9–16) were generated by reversing Lists 1–8 from top-to-bottom, with the exception of the 12 fillers at the beginning of the lists. Each of the experimental lists was presented to four participants, each of whom received only one list.

#### Normative Ratings

Normative ratings were collected on all experimental items. In addition to notional number, which was of *a priori* interest, imageability and semantic integration ratings were also collected because these factors have been shown to affect subject–verb agreement (Eberhard, 1999; Solomon and Pearlmutter, 2004).

The imageability and semantic integration ratings were collected from 80 undergraduates at the University of Illinois who participated in this study or in other language experiments, after the completion of all sentence completion tasks. For these ratings, each rater received 40 items from two experimental lists, plus eight additional fillers. Additional lists were constructed with the conjoined NPs in their flipped order (e.g., *the milk and cheese* →*the cheese and milk*). In compiling the ratings, the scores for the two different word orders were averaged together.

For the imageability ratings, participants were instructed to rate items on a scale of 1 (low imageability) to 7 (high imageability) based on how easy they were to picture. Example items of low imageability (e.g*., the truth of the matter*) and high imageability (e.g., *the skyscraper in the city*) were given. For the semantic integration ratings (Solomon and Pearlmutter, 2004), participants rated items on a scale of 1 (not linked) to 7 (closely linked) based on how closely the two items were linked. Examples of closely linked items (e.g., *the cookies with chocolate chips*) and non-linked items (e.g., *the cookies with milk*) were given. The notional number ratings were completed by 84 independent raters via Amazon Mechanical Turk using the same lists as were compiled for the imageability and semantic integration norms. Participants rated how likely, on a scale of one to five, they would be to refer to the NPs as *it* (1) or *they* (5). The phrase *heat and humidity* was given as an example of something that could be replaced with either pronoun. We used pronoun ratings, as pronoun agreement has been shown to closely reflect the notional number valuation (Bock et al., 2004), and because other types of ratings (e.g., “Does this refer to ‘one thing’ or ‘many things’?”), which have been used with success for distributive NPs (Lorimor et al., 2008), seemed to confuse participants in pilot studies, as conjoined NPs specifically name two things.

Because we collected three different types of ratings on each noun phrase, which could each serve as predictors in our statistical model, we conducted tests for multicollinearity using the variance inflation factor (Vif) (Midi et al., 2010). All Vif factors were lower than 1.7, which is below the lowest threshold suggested by Midi et al. (2010) for weaker models (2.5). Therefore, we included all of the predictor variables from norming data in our statistical models.

#### Procedure

Preambles were presented auditorily using PsyScope 1.2.5 (Cohen et al., 1993). Participants were tested individually. They were seated in front of a Macintosh computer, wearing a head-mounted microphone for voice recording. Participants were instructed to listen to each phrase (preamble), to repeat each phrase exactly as they heard it, and then to complete the sentence, speaking as quickly as possible. No practice trials were given. However, fillers at the beginning of each list served as a covert practice phase. Feedback occurred if participants changed the words in the preamble or failed to complete the sentence, but no feedback was given about the form of the verb used. The experimenter advanced through trials with a mouse click. Sessions lasted approximately 10 min and were recorded on a digital recorder.

#### Scoring

All responses were transcribed and scored. Responses were considered valid if participants correctly repeated the preamble and completed the sentence, using a number-marked verb, with no intervening material. All other responses were scored separately. Responses were excluded if the participant inserted intervening material between the preamble and the verb, generally in the form of a prepositional phrase modifying the subject (e.g., *The name and address of the applicant is/are…*). Responses were also excluded if participants produced a verb that was unmarked for number, if there was a misrepetition of the preamble, or if no completion was provided.

Overall, there were 1,280 responses for the experimental items, 664 (52%) of which were valid responses with verbs that were marked as singular or plural. Within the valid items, 292 (44%) singular and 372 (56%) plural verbs were produced. The raw numbers and proportion of plural verbs produced by category for all valid responses are listed in **Table 3**.

**Table 3:** Distribution of responses by noun type and percentage of plural versus singular verbs for non-miscellaneous responses in the oral sentence completion task (Experiment 1).

Conjunct type	Singular verbs (% singular)	Plural verbs (% plural)	Miscellaneous responses
Count	111 (43%)	146 (57%)	255
Mass	126 (55%)	105 (45%)	281
Collective	55 (31%)	121 (69%)	80

### Results

The data were analyzed using Bayesian mixed effects modeling (Nicenboim and Vasishth, 2016; Sorensen et al., 2016) using the rstanarm package version 2.15.4 (Stan Development Team, 2017) in R version 3.4.0 (R Core Team, 2017) with default, weakly informative priors. Bayesian methods, which are becoming increasingly popular in psychology (Vandekerckhove et al., 2018), have several advantages over mixed effects models. For example, Bayesian models, such as those implemented in rstanarm, avoid the problems of convergence that are frequently encountered in mixed effects models (Eager and Roy, 2017). In addition, Bayesian models provide a natural way of evaluating probability and the strength of evidence, given existing data and prior beliefs (Etz and Vandekerckhove, 2017).

The model run in rstanarm returns estimates and credible intervals for each parameter using nearly identical syntax to lme4 (Bates et al., 2015). Default priors are centered at zero, normally distributed, and with standard deviations (scales) of 10 for the intercept and 2.5 for regression coefficients (Stan Development Team, 2017). The dependent variable, verb number, was entered using “0” for singular, “1” for plural. Fixed effects included the categorical variable of noun type, with count NPs as the baseline, as these were most likely to refer to two distinct entities, rather than masses or groups, and are therefore the most canonical examples of conjoined NPs. Normative ratings for imageability, semantic integration, and notional number were also entered as fixed effects (centered at their sample means), as were two-way interactions for each of the normative ratings with categorical noun type. The model also included random intercepts by item and random slopes (for noun type) by participant. The model summary with credible intervals is shown in **Table 4**. The table also includes the *R*^∧^ statistic, which is a ratio of between/within chain convergence; if all *R*^∧^-values are <1.1, this meets the conventional rule for convergence (Gelman et al., 2013).

**Table 4:** Parameter estimates, quantiles and the *R*^∧^ statistic from the mixed logit model run with rstanarm on Experiment 1.

Fixed effects	Mean	2.5%	97.5%	*R*^∧^
**(Intercept)**	**0.629**	**0.530**	**0.729**	**1.005**
Conjunct type: collectives	0.104	–0.179	0.397	1.003
**Conjunct type: mass**	–**0.147**	–**0.287**	–**0.009**	**1.002**
**Notional number**	**0.267**	**0.051**	**0.487**	**1.001**
**Imageability**	**0.156**	**0.082**	**0.229**	**1.005**
Semantic integration	–0.080	–0.184	0.021	1.004
Conjunct type: collectives × Notional number	–0.311	–0.664	0.039	1.001
Conjunct type: mass × Notional number	–0.205	–0.562	0.137	1.002
Conjunct type: collectives x Imageability	–0.176	–0.380	0.026	1.003
Conjunct type: mass × Imageability	–0.090	–0.182	0.003	1.005
Conjunct type: collectives × Semantic integration	0.036	–0.115	0.201	1.005
Conjunct type: mass × Semantic integration	0.025	–0.136	0.192	1.002

*Ninety-five percent credible intervals that do not include zero are bolded*.

Consistent with Nicenboim and Vasishth (2016), we consider there to be strong evidence for an effect of a parameter if its 95% credible interval does not include zero. As seen in **Table 4**, there was strong evidence that conjoined NPs involving mass nouns differed from baseline (count nouns), but there was no evidence that conjoined NPs containing collective nouns were different from those containing count nouns. In addition to these effects of conjunct type, there was also strong evidence for main effects of notional number and imageability. The positive direction for the notionality and imageability parameters suggests that preambles that were more imageable or that were more notionally plural were more likely to lead to plural agreement.

### Discussion

Overall, these results show that both noun type and notional number impact agreement with conjoined NPs. Bayesian mixed effect logistic regression models, which included both categorical predictors of conjunct type and continuous predictors based on norming data, showed that conjunct type was an important predictor of agreement, as conjoined mass nouns showed more singular agreement than conjoined count nouns. Notional number also played a role, as more notionally singular items took more singular agreement. Similarly, conjoined NPs that were less imageable took more singular agreement than the more imageable items.

**FIGURE 1:**
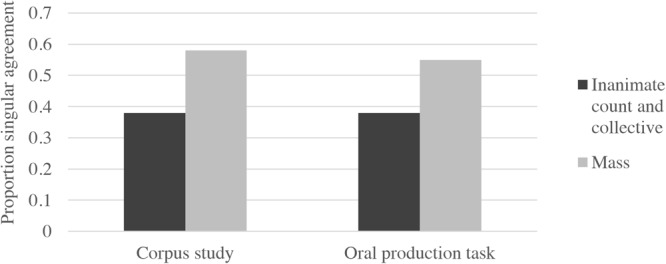
Proportion singular verbs in the corpus study and oral production task (Experiment 1).

The results of the corpus study and the oral production task thus provide converging evidence that singular agreement is common with conjoined NPs, especially if both of the conjoined nouns are singular and inanimate, as the proportions of singular agreement in the oral production task are nearly identical to those reported for conjoined singular, inanimate nouns in the corpus analysis. In **Figure 1**, we show the proportion of singular verbs in our corpus study and Experiment 1. For the purposes of directly comparing the corpus study to Experiment 1, we only included conjoined NPs from the corpus study that had inanimate, singular nouns. We also collapsed across count and collective nouns in Experiment 1, as there was only a single category for count/collective nouns in the corpus task.

Therefore, given the frequency of singular agreement with conjoined NPs both in the laboratory and in our internet-based corpus, we can conclude that singular agreement with conjoined NPs is normative and is influenced by notional number, at least for NPs that are composed of inanimate or mass nouns. At this point, there are three potential explanations for how agreement is computed with conjoined NPs. One possibility is that conjoined NPs have no grammatical specification, and that, when both of the conjoined nouns are singular, any plural agreement would be due to notional information. Another possibility is that individual conjoined NPs do have discrete singular or plural specifications that may differ from one individual to the next, such that they look probabilistic at a population level, similar to the account for collectives proposed in Bock et al. (2006). A third possibility is that conjoined NPs do have a grammatical specification that influences agreement alongside notional information, but instead of the grammatical specification being a discrete, binary feature, it is probabilistic and reflects the distributional information in the language.

In order to arbitrate between these competing explanations, we performed a written production task that involved a learning component. If there is an intermediate grammatical number specification for conjoined NPs (i.e., a number specification that is somewhere between singular and plural) that is sensitive to distributional information, then it should be possible to shift that specification along the continuum between singular and plural through an implicit learning task. If this is the case, then experience with plural verbs agreeing with one set of conjoined NPs in a biasing task should lead to more plural verb agreement with a new set of conjoined NPs in a later task. However, if conjoined NPs do not have probabilistic, intermediate grammatical number specifications, so that any flexibility in agreement is determined only by notional effects on agreement, then it should not be possible to bias participants toward more singular or plural agreement. Similarly, we would not expect participants to adjust their rates of singular or plural agreement with conjoined NPs after a biasing task if grammatical number valuations are only probabilistic at the population level, where some or all tokens of a type of agreement controller are specified as singular for some speakers and plural for others.

## Written Production Task (Experiment 2a)

Previous work has shown that, through manipulating participants’ short-term experience with particular constructions, it is possible to adjust participants’ patterns of verb agreement (Haskell et al., 2010), which can only happen if the grammar contains a number specification that can be adjusted. This provides a natural opportunity to test whether conjoined NPs have a probabilistic grammatical number specification that is shaped by distributional information. If there is indeed a probabilistic grammatical specification on conjoined NPs, we would expect that, if participants encounter sentences containing conjoined NPs and plural verbs in a biasing story, they would subsequently use more plural verb agreement with conjoined NPs, compared to participants who encountered conjoined NPs with singular or unmarked verbs. Alternatively, if participants do not have probabilistic number valuations for conjoined NPs that they update based on experience, then we would not expect verb agreement with conjoined NPs to be shaped by co-occurrence information.

### Method

#### Participants

One hundred and twenty-one participants were Amazon Mechanical Turk workers with IP addresses in the United States. Participants received a small payment for their completion of the study. This study was carried out in accordance with the recommendations of the Institutional Review Board at Bucknell University. Participants indicated their informed consent by clicking on the link that initiated the study. The protocol was approved by the Institutional Review Board at Bucknell University.

#### Materials and Procedure

The experiment was administered using the online survey software Qualtrics, following the procedures outlined in Haskell et al. (2010), with the exception that we added a baseline task before the biasing story so that we could compare individual participants’ agreement patterns before and after the biasing story. In our experiment, participants first completed the baseline task, then read a biasing story that paired conjoined NPs with singular, plural, or unmarked verbs, and then completed sentences in the “story completion” portion of the task. For the baseline and story completion tasks, all of the experimental items consisted of conjoined NPs with an embedded prepositional phrase. All of the nouns in the conjoined NPs were singular mass or deverbal nouns, and the nouns in the embedded prepositional phrase were all singular (e.g., “*the principle and interest for the loan*”).

The baseline task consisted of 8 items as described above and 24 filler items (complex NPs, nouns with adjectival modifiers, and plural conjoined NPs), 16 of which were singular, and eight of which were plural. All participants received the same baseline items (“fragments”) in a randomized order. Participants were told that the fragments were the beginnings of sentences and that they should provide sentence completions for each of the fragments they saw. The conjoined NPs were followed by prepositional phrases with singular local nouns so that participants would be more likely to produce a verb directly after the preamble, rather than their own prepositional phrase (e.g., “the principle and interest …*for the student loans*”), which might contain either singular or plural nouns and increase the variability in the types of sentences produced.

Following the baseline task, we presented participants with a story task, which attempted to bias participants toward singular or plural verb agreement with conjoined NPs. In the story task, participants first read a “biasing” story about preparations for a trade show, and then in the completion portion, they completed 32 sentences based on the fragments they were given. Similar to Haskell et al. (2010), the story was composed of primes, counterbalance sentences, and fillers. Examples of prime and counterbalance sentences are provided in **Table 5**.

**Table 5:** Sample story prime and counterbalance sentences (Experiments 2a and 2b).

Prime	Singular	The tea and coffee was spread out on a long table
	Plural	The tea and coffee were spread out on a long table
	Unmarked	The tea and coffee had been spread out on a long table
Counterbalance	Singular	But the ice cream sundaes were still in the freezer
	Plural	But the ice cream was still in the freezer
	Unmarked	But the ice cream sundaes stayed in the freezer

Eight sentences in the biasing story contained a conjoined NP comprised of singular mass or deverbal nouns, and some of the sentences had an embedded prepositional phrase containing a singular noun when it made sense based on the sentence context. These sentences were the “story prime” sentences. As we were interested in whether exposure to singular or plural verbs with conjoined NPs would change participants’ agreement patterns with conjoined NPs, we had three prime conditions: singular, plural, and unmarked. In the singular-prime condition, the conjoined NPs were paired with singular verbs. In the plural-prime condition, the conjoined NPs were paired with plural verbs. In the unmarked-prime condition, the conjoined NPs were paired with verbs that were unmarked for number. There were also 8 counterbalance sentences in the story prime task, which were included so that each participant would encounter an equal number of singular or plural verbs overall (e.g., the “singular” counterbalance contained a plural subject NP and a plural verb). Therefore, if participants produced more plural agreement with conjoined NPs in response to plural primes, it was not because they had been flooded with plural verbs, but that they had learned that plural verbs co-occur with conjoined NPs (in the plural prime condition). All other verbs in the story and completion task that were not in the story prime/counterbalance pairs, including those in fillers, were unmarked for number. The participants in the unmarked-prime condition received only unmarked verbs throughout the whole experiment. The complete set of items for the baseline task, biasing story, and completion task is in the Supplementary Materials.

In the completion portion of the story task, participants were instructed to type a logical completion for each of the fragments, given the story they had just read. As in Haskell et al. (2010), the sentence fragments were divided into four types: counterbalance, prime, filler, and target; participants completed 8 sets of sentences, with each set containing one sentence of each type, always presented in the same order (see **Table 6**). In the singular-prime condition, a participant first encountered a plural verb in the counterbalance sentence, then a conjoined NP with a singular verb in the prime sentence, followed by an unmarked verb in the filler sentence. For each of these sentences, the completion portion began after the verb. Then, the target sentence was a conjoined NP subject with an embedded PP containing a singular local noun, and participants provided the verb in their completions. None of the nouns in any of the baseline items, story, or completion task were duplicated across tasks. However, the items in the baseline and story completion tasks were matched, so that the conjoined NPs in the baseline and the story completion tasks had equal numbers of mass and deverbal nouns and the same prepositions (*by, of, in*…).

**Table 6:** Sample set of items from the story completion task (Experiments 2a and 2b).

Counterbalance	Singular	Out in the exhibition hall, people were shouting over…
	Plural	Out in the exhibition hall, someone was shouting over…
	Unmarked	Out in the exhibition hall, someone had been shouting over…
Prime	Singular	The speed and capacity of the new prototype was being questioned by…
	Plural	The speed and capacity of the new prototype were being questioned by…
	Unmarked	The speed and capacity of the new prototype had been questioned by…
Filler		During the demonstration, the new machine did not lift…
Target		The exaggeration and lying by the company…

#### Scoring

Responses from both the baseline and story completion task were scored as having singular verbs, plural verbs, or “other” – including verbs that were unmarked for number. Out of the 121 participants, we excluded anyone who did not have at least 4 number-marked verbs in both the baseline task and in the story completion task, (12 in plural-prime, 12 in singular-prime, and 7 in unmarked-prime conditions), leaving 90 participants (30 in each prime-condition).

Overall, from these 90 participants, there were 1440 responses on the experimental items, 720 for the baseline items, and 720 for the story-completion items. For the baseline items, 501 (70%) were valid responses with verbs that were marked as singular or plural. Within these valid items, 446 verbs (89%) were singular and 55 (11%) were plural. For the story completion items, 526 (73%) were valid responses with verbs that were singular or plural. Within these valid items, 462 verbs (88%) were singular, and 64 (12%) were plural. The raw numbers and percentage of singular and plural verbs produced by category for all valid responses are listed in **Table 7**.

**Table 7:** Distribution of responses by prime type and percentage of singular versus plural verbs for baseline and story completion tasks (Experiment 2a).

Prime type	Task	Singular verbs (% singular)	Plural verbs (% plural)	Miscellaneous responses
Singular	Baseline	145 (88%)	20 (12%)	75
	Story	150 (89%)	19 (11%)	71
Plural	Baseline	153 (91%)	15 (9%)	72
	Story	135 (80%)	34 (20%)	71
Unmarked	Baseline	148 (88%)	20 (12%)	72
	Story	177 (94%)	11 (6%)	52

### Results

As in Experiment 1, the data were analyzed using Bayesian mixed effects modeling (Nicenboim and Vasishth, 2016; Sorensen et al., 2016) using the rstanarm package version 2.15.4 (Stan Development Team, 2017) in R version 3.4.0 (R Core Team, 2017) with default, weakly informative priors. After the first model had five divergent transitions, we set *adapt_delta* to 0.99 in order to avoid divergent transitions, which reduces the step size and therefore requires more steps to explore the posterior distribution (Stan Development Team, 2017). The dependent variable verb number was entered as “0” for singular, “1” for plural. Prime type was entered as a 3-level fixed effect, with the unmarked-prime as the reference. Task (baseline vs. story-completion) was also entered as a fixed effect, with the baseline task entered as the reference. The model included both task and prime type as fixed effects, as well as their interaction. We entered participants and items as random intercepts and random slopes for prime condition by item. The model summary with parameter estimates, 95% credible intervals, and *R*^∧^*-*values as diagnostics of model convergence is shown in **Table 8**.

**Table 8:** Parameter estimates, quantiles, and the *R*^∧^ statistic from the mixed logit model run with rstanarm on Experiment 2a.

Fixed effects	Mean	2.5%	97.5%	*R*^∧^
**(Intercept)**	**0.11**	**0.03**	**0.2**	**1**
Prime: plural	–0.03	–0.13	0.07	1
Prime: singular	0.02	–0.08	0.11	1
Task: story	–0.06	–0.17	0.06	1
**Prime: plural × Task: story**	**0.16**	**0.03**	**0.29**	**1**
Prime: singular × Task: story	0.03	–0.07	0.14	1

*Ninety-five percent credible intervals that do not include zero are bolded*.

The credible intervals shown in **Table 8** suggest that, although there was no main effect of prime-type or task, there was strong evidence for an interaction between prime and story, such that the increase in plural verb agreement was greater in the plural condition than in the unmarked condition, providing evidence for priming. However, there was no evidence of singular priming, i.e., a decrease in plural verbs with conjoined NPs from baseline to the story completion task in the singular-prime condition.

As an additional measure, and to gain more confidence that the observed priming effect was due to an increase in plural agreement in the plural-prime condition and not to any changes in the unmarked condition, we ran paired *t*-tests on an analysis by participants that compared the proportion of plural responses in the baseline task to the proportion of plural responses in the story completion task, which were one-tailed for the singular and plural prime conditions, and two-tailed for the unmarked prime condition. Results showed that there was a significant difference between the baseline and story completion task in the plural-prime condition [*t*(29) = 3.35, *p* < 0.01], but not in the singular-prime condition [*t*(29) = -0.44, *p* > 0.33] or the unmarked-prime condition (*t*(29) = -1.45, *p* > .15). This analysis provided additional support that the effects reported in the Bayesian analysis are due to a change in the plural-prime condition and also for an asymmetry between plural versus singular priming.

### Discussion

These results show that participants in the plural-prime condition increased their rate of plural agreement with conjoined NPs from baseline to the story completion task, relative to the unmarked-prime condition. This provides evidence, consistent with Haskell et al. (2010), that language users are responsive to the distributional patterns in their environment and that they adjust their production behavior in response to recent input.

The fact that participants kept track of this distributional information (i.e., how often conjoined NPs occurred with plural verbs) and used that to adjust their own agreement patterns in the written completion task provides evidence that conjoined NPs have probabilistic grammatical number specifications. As there was no overlap in the nouns used in the baseline phrases, the story phrases, and the completion phrases, this suggests that participants in the plural-prime condition were responding to the co-occurrence of conjoined NPs with plural verbs in the biasing story, rather than exhibiting episodic memory of individual subject–verb pairs.

We observed an asymmetry in priming between the plural-prime condition, which showed an increase in plural agreement after plural primes, and the singular-prime condition, which did not show a decrease in the rate of plural agreement when conjoined NPs were paired with singular verb forms. The same asymmetry was reported by Haskell et al. (2010), who found that participants shifted their agreement patterns after plural verb primes, but did not shift their behavior when the collective NPs were paired with singular verbs. One possible explanation for this asymmetry has to do with frequency of verb forms. The Corpus of Contemporary American English (Davies, 2008), which provides word form frequencies, contains more than twice the number of singular verb forms “is” and “was,” compared to their plural counterparts. Less frequent structures are frequently considered to be more “marked”; when more marked structures are encountered, this may lead to greater priming. An alternate explanation for the lack of an effect of singular primes is that the rate of plural agreement with these conjoined NPs was already quite low, leading to a floor effect in the singular-prime condition.

## Written Production Task With Preamble Repetition (Experiment 2b)

Given that, until now, there have only been a few studies (including Experiment 2a, Haskell et al., 2010 and Lorimor et al., 2016a) that have shown participants shifting agreement patterns based on distributional input, we sought to replicate the results of Experiment 2a, with one small change. In Experiment 2a, participants only typed the completion of the sentence. This differs from many oral production tasks, which require that participants repeat the preamble before completing the sentence, as preamble repetition allows experimenters to verify that participants correctly encoded the preamble.

The repetition plus completion procedure would also allow us to determine whether repetition of the preamble might change the degree to which participants adjusted their agreement behavior in response to distributional information. On one hand, typing the preamble might increase participants’ attention toward the subject nouns themselves and reinforce previous biases about subject–verb agreement, thus preventing participants from updating their number specifications for conjoined NPs. On the other hand, re-typing the preamble might lead to greater adjustments in participants’ agreement patterns, since participants were typing both subjects and verbs, which might reinforce the relationship between the subject and the verb.

### Method

#### Participants

One hundred sixty-two participants were recruited through Amazon Mechanical Turk and received a small payment for their completion of the survey. The survey was limited to Mechanical Turk workers who had IP addresses in the United States and who had not participated in the previous study. This study was carried out in accordance with the recommendations of the Institutional Review Board at Bucknell University. Participants indicated their informed consent by clicking on the link that initiated the study. The protocol was approved by the Institutional Review Board at Bucknell University.

#### Materials and Procedure

The materials and procedure were identical to those in Experiment 2a, with one exception. In Experiment 2a, participants were only instructed to provide sentence completions for the fragments they saw. In Experiment 2b, participants were instructed to retype the fragment and then to provide a sentence completion.

#### Scoring

Like Experiment 2a, responses from both the baseline and story completion task were scored as having singular verbs, plural verbs, or “other” – including verbs that were unmarked for number. In Experiment 2b, we also excluded responses that modified the preamble, usually by adding additional words (e.g., *the corruption and extortion of the politician to protect his family is still unjustifiable.*) Out of the 162 participants, we excluded anyone who did not have at least 4 usable responses that repeated the preamble exactly and included number-marked verbs in both the baseline task and in the story completion task (28 participants in the plural-prime, 21 in the singular-prime, and 23 in the unmarked-prime conditions), leaving 90 participants (30 in each prime-condition).

Overall, from these 90 participants, there were 1440 responses, 720 for the baseline items, and 720 for the story-completion items. For the baseline items, 498 (69%) were valid responses with verbs that were marked as singular or plural. Within these valid items, 435 verbs (87%) were singular, and 63 (13%) were plural. For the story completion items, 492 (73%) were valid responses that were marked as singular or plural. Within these valid items, 401 verbs (82%) were singular, and 91 (18%) were plural. The raw numbers and percentage of plural verbs produced by category for all valid responses are listed in **Table 9**.

**Table 9:** Distribution of responses by prime type and percentage of singular versus plural verbs for baseline and story completion tasks (Experiment 2b).

Prime type	Task	Singular verbs (% singular)	Plural verbs (% plural)	Miscellaneous responses
Singular	Baseline	138 (82%)	31 (18%)	71
	Story	140 (83%)	29 (17%)	71
Plural	Baseline	139 (91%)	13 (9%)	88
	Story	123 (72%)	49 (28%)	68
Unmarked	Baseline	158 (89%)	19 (11%)	63
	Story	138 (91%)	13 (9%)	89

### Results

The data were analyzed as in Experiment 2a. The dependent variable verb number was entered as “0” for singular, “1” for plural. Prime type was entered as a 3-level fixed effect, with the unmarked-prime as the reference. Task (baseline vs. story-completion) was also entered as a fixed effect, with the baseline task entered as the reference. The model included both task and prime type as fixed effects, as well as their interaction. We entered participants and items as random intercepts and random slopes for prime condition by item. The model summary with parameter estimates, 95% credible intervals, and *R*^∧^-values as diagnostics of model convergence is shown in **Table 10**.

**Table 10:** Parameter estimates, quantiles, and the *R*^∧^ statistic from the mixed logit model run with rstanarm on Experiment 2b.

Fixed effects	Mean	2.5%	97.5%	*R*^∧^
**(Intercept)**	**0.11**	**0.01**	**0.20**	**1**
Prime: plural	–0.02	–0.12	0.08	1
Prime: singular	0.08	–0.02	0.18	1
Task: story	–0.03	–0.16	0.09	1
**Prime: plural × Task: story**	**0.21**	**0.09**	**0.33**	**1**
Prime: singular × Task: story	0.00	–0.12	0.12	1

*Ninety-five percent credible intervals that do not include zero are bolded*.

**FIGURE 2:**
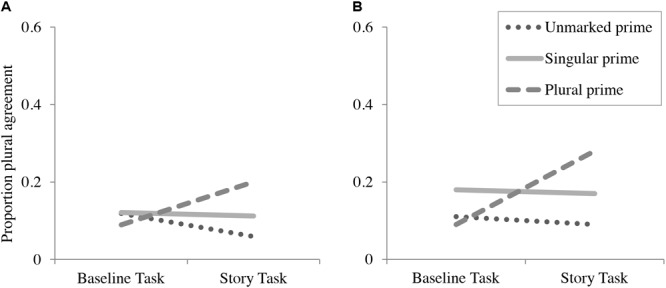
Proportion plural verbs for conjoined noun phrases (NPs) by prime type in baseline and story completion tasks in completion-only task **(A)** and preamble repetition task **(B)**.

The results for Experiment 2b are similar to Experiment 2a. The 95% credible intervals shown in **Table 10** suggest that, like Experiment 2a, there was no main effect of prime-type or task, but that there was an interaction between prime and task, such that the increase in plural verb rate, compared to baseline, was greater in the plural-prime condition than in the unmarked-prime condition. Like Experiment 2a, there was no effect of the singular prime on the story completion task. As an additional measure, like in Experiment 2a, we conducted paired *t*-tests based on an analysis by participant that compared the proportion of plural agreement in the baseline task to the story completion task, using the same procedures as described for Experiment 2a. The results showed a significant difference between the baseline and story completion task in the plural-prime condition [*t*(29) = 4.58, *p* < 0.001], but no significant difference in the singular-prime condition [*t*(29) = -0.33, *p* > 0.37] or the unmarked-prime condition [*t*(29) = -0.41, *p* > 0.69]. **Figure 2** illustrates the pattern of results for both Experiments 2a and 2b.

### Discussion

The patterns of results were similar between Experiments 2a and 2b, as participants increased their rate of plural agreement with conjoined NPs after the plural biasing story in both the completion-only (Experiment 2a) and the preamble repetition (Experiment 2b) tasks. The overall rates of plural agreement were similar between Experiments 2a and 2b, suggesting that repetition of the preamble itself does not substantially change participants’ patterns of agreement with conjoined NPs.

Experiment 2b thus provides further evidence that participants will adjust their agreement patterns based on experience and that conjoined NPs have malleable number specifications that are responsive to this input. It is important to note that these experiments, like Haskell et al. (2010), included counterbalance sentences such that participants in each of the singular and plural prime conditions encountered equal numbers of singular and plural verbs. Therefore, the increase in plural agreement in the plural-prime condition was not due to participants having been flooded by plural verbs. Instead, participants in the plural-prime condition encountered conjoined NPs that agreed with plural verbs, and the exposure to that distributional information was sufficient to increase participants’ rates of plural agreement with conjoined NPs in the story completion task.

Thus, Experiments 2a and 2b provide evidence that conjoined NPs have phrasal-level grammatical specifications, and that these number specifications are sensitive to distributional input. This is consistent with the view that grammatical representations can be probabilistic (Lau et al., 2017), rather than discrete, and that distributional information shapes grammatical number representations, consistent with the Constraint Satisfaction model (Haskell et al., 2010).

One potential counter-argument is that, instead of changing participants’ grammatical number specifications, we might have been changing their notional valuations of the conjoined NPs in the task. Therefore, if participants were in the plural-prime condition, then the fact that they encountered plural verbs with conjoined NPs during the story completion task might have led them to conceptualize the conjoined NPs as more plural, compared to the participants in the unmarked-prime or singular-prime condition. However, if participants were changing their conceptual representations, then we would expect to see an increase in singular agreement in the singular-prime condition, which we did not see. We also have evidence from a number of sources, including Bock et al. (2006), that verb agreement patterns much more closely with grammatical, rather than notional number valuations. For example, Bock et al. (2006) showed that speakers of British English and American English had similar notional number valuations for collective nouns like *team*, even though British English speakers were more likely to say *The team are…*, while American English speakers would be more likely to say *The team is….* This difference across dialects in grammatical, but not notional, number provides support that notional and grammatical number are distinct and that speakers can maintain separate representations of each.

## General Discussion

The results of the corpus analysis, oral production task, and written production tasks can be summarized as follows: (1) Conjoined subjects regularly agree with both singular and plural verbs; (2) Conjoined NPs with enhanced notional plurality are more likely to take plural agreement than conjoined NPs that are more notionally singular; and (3) Agreement with conjoined NPs is sensitive to distributional information, as participants in both written production tasks used more plural verbs in the story completion task in the plural-prime condition, compared to baseline, than those in the unmarked-prime condition.

Altogether, these experiments provide evidence that, while conjoined NPs do often reflect notional information, they are not “number-less,” and that they do not carry discrete plural or singular number values. Instead, we propose that conjoined NPs do have a grammatical number specification, but that this grammatical specification is probabilistic and reflects the distributional patterns in the environment.

### Models of Agreement

These results have important implications for theoretical models of agreement. In models of agreement that are based on binary, discrete number representations, nouns themselves have either singular or plural specifications, which are then communicated to verbs (Levelt et al., 1999). The Marking and Morphing model (Eberhard et al., 2005) maintains this discrete view of lexical items, but incorporates feature percolation from the items within the subject NP and allows for probabilistic verb selection based on the grammatical and notional information in the subject NP. The Marking and Morphing model also allows for probabilistic lexical specifications at the population level (Bock et al., 2006), which allows the model to maintain the view that grammatical values are discrete, but that for a class of nouns, some nouns for some speakers may be plural while others are singular. Thus, some lexical classes, such as collective nouns, behave like they have intermediate number specifications.

Up until this point, it has been unclear how Marking and Morphing (Eberhard et al., 2005) can account for the variability in agreement patterns with conjoined NPs. Several papers, including Bock et al. (2004) and Bock and Middleton (2011), have used conjoined NPs as examples of instances in which notional number can determine agreement, which implies that the Marking and Morphing account might predict that conjoined NPs do not have any grammatical specifications on their own, apart from any lexical specifications within the NP. This is also consistent with the idea that the Marking and Morphing model does not incorporate any phrasal-level grammatical specifications. However, given that Marking and Morphing does have the capacity to include functionally probabilistic grammatical values for a class of agreement controllers, Lorimor et al. (2016b) used that capability to model agreement with conjoined NPs in Dutch using the mathematical formula provided by the Marking and Morphing model (Eberhard et al., 2005). In their model implementation, Lorimor et al. (2016b) obtained an intermediate, probabilistic grammatical number value based on how often conjoined NPs co-occurred with plural verbs in a Dutch corpus analysis, and they set that probabilistic number value on the ‘and’ within their implementation of the Marking and Morphing model. Therefore, while the assumptions of the Marking and Morphing model are that grammatical number values are discrete and that only lexical items have grammatical number features, the model does have the capabilities of incorporating probabilistic number values, even at the level of the individual speaker, and of including phrasal-level specifications if those can be specified on a particular lexical item.

On the other hand, the Constraint Satisfaction model (Haskell et al., 2010; Haskell and MacDonald, 2003) specifically predicts that grammatical values should be probabilistic and should vary in response to distributional input. In contrast to Marking and Morphing (Eberhard et al., 2005), the Constraint Satisfaction model allows for statistical learning at the phrasal level, so if a particular construction (e.g., conjoined NP) frequently co-occurs with a plural verb, this would increase the degree to which conjoined NPs activate plural verbs during sentence planning because of the construction in the subject NP, and beyond any activation stemming from the individual lexical items involved. Haskell et al. (2010) argue that the priming mechanisms associated with short-term changes, such as those observed in Experiments 2a and 2b, are not unlike the learning mechanisms that confer long-term changes to agreement patterns. The changes seen in subject–verb agreement, based on biasing stories, might be cumulative and persistent, just as syntactic priming effects are (Kaschak et al., 2011; Tooley and Traxler, 2018). Haskell et al.’s (2010) proposal thus ties together several language production phenomena, including structural priming and agreement, under the same umbrella of implicit learning. In the present study, we only measured short-term priming effects, and we acknowledge a need for future research to directly link these short-term priming effects in agreement to longer-term changes in the language production system.

### Intermediate Number Specifications Versus Optional Closest Conjunct Agreement

Thus, our proposal is that intermediate, phrasal-level number specifications on the conjoined NP combine with notional information to jointly produce either singular or plural agreement, and that the number specifications on conjoined NPs arise through implicit learning. An alternative explanation for why singular verbs would sometimes appear with conjoined NP subjects is closest conjunct agreement, in which the verb agrees with the closest noun, rather than with the conjunction as a whole. This is the explanation that Keung and Staub (2016) gave, based on their participants’ tendency to agree with the closest noun, and it may account for some of the singular agreement in these data, especially with the conjoined count nouns. However, closest conjunct agreement [via cue-based “mis-retrieval” (Keung and Staub, 2016)] is rare cross-linguistically for preverbal subjects (Willer Gold et al., 2017), and it does not predict the sensitivity to the range of notional information that we observe in the English data. Indeed, if singular agreement with conjoined NPs is just closest conjunct agreement, then it would be hard to explain why singular agreement was so common for mass nouns, such as *the copper and iron*, since, *a priori*, there is no reason that participants should be more likely to forget that there were two nouns in that subject NP, compared to other conjoined NPs such as *the viola and violin*.

In fact, we would argue that notional number impacts agreement separately from cue-based retrieval, and that the variability in agreement with conjoined NPs is not primarily due to errors in retrieving the full subject NP. Lorimor et al. (2016b) explored the relationship between notional number agreement and cue-based retrieval with conjoined NPs in Dutch and German. They showed that both Dutch and German showed notional effects on agreement (more singular agreement with deverbal/mass NPs than with count/animate NPs). Further, they showed that these effects of notionality were separate from an effect of grammatical gender, in which participants produced more singular agreement when the conjoined nouns had the same gender, compared to when the nouns had different genders. This effect of more singular agreement with same-gender nouns is consistent with a cue-based retrieval model of agreement, since the speakers would not have distinct gender cues that would help them retrieve both of the nouns in the subject NP. However, as there was no interaction between notionality and grammatical gender, cue-based retrieval did not explain notional effects in either the Dutch or the German data, and we do not believe that it explains the full pattern of singular agreement in these English data, either.

### The Specificity of Distributional Information

In Experiments 2a and 2b, we demonstrate that speakers adjust their agreement patterns with conjoined NPs based on their experience reading sentences involving agreement with conjoined NPs, and we argue that this is evidence that speakers are updating the grammatical number specifications of conjoined NPs. One question that remains, however, is how fine-grained speakers’ grammatical specifications are. Specifically, are speakers learning about the co-occurrence of singular verbs with inanimate/abstract conjoined NPs and setting grammatical specifications for mass or inanimate conjoined NPs separately from their specifications for count/animate conjoined NPs? While this remains an open question to some degree, we do have evidence that speakers generalize their learning across classes of nouns and across constructions. In Haskell et al. (2010), not only did they find that collective-plural primes led to more plural verbs with collective NPs (Experiment 1), but they also found that those same biasing stories led participants to produce more plural verbs with non-collective NPs (Experiment 2). Similarly, Lorimor et al. (2016a) found generalization from “All of the…” constructions to “Each of the…” constructions, such that participants produced more plural verbs for sentences beginning with “Each of the Xs…” after having encountered a series of “All of the…” constructions paired with plural verbs. Therefore, while more systematic investigation of the magnitude of within-construction and between-construction learning is warranted, we do have evidence that speakers generalize their learning about the co-occurrence of subject NPs and singular or plural verbs to related classes of nouns and related constructions.

## Conclusion

This study provides an empirical analysis of agreement with conjoined NP subjects in English and shows that speakers use both singular and plural verbs with conjoined NPs in English. Furthermore, we show that, while notional number plays an important role in agreement with conjoined NP subjects, conjoined NPs themselves are not “number-less”. Instead, we argue that they contain intermediate, probabilistic grammatical number specifications that change based on distributional information. This provides additional evidence that speakers keep track of distributional information, even for structures like conjoined NPs, and provides support for the proposals in Haskell et al. (2010), which link agreement to structural priming and other types of implicit learning.

## Data Availability

All datasets analyzed for this study can be found in the Open Science Framework Repository at https://osf.io/sr74n/.

## Author Contributions

HL and EM conceptualized the experimental design for Experiment 1 and wrote the manuscript. HL collected, transcribed, and coded the data, performed the corpus analysis, and performed the statistical analyses. HL and NA conceptualized the experimental design for Experiments 2a and 2b. NA collected, transcribed, and coded the data. All authors critically reviewed the manuscript and approved the final version for publication.

## Conflict of Interest Statement

The authors declare that the research was conducted in the absence of any commercial or financial relationships that could be construed as a potential conflict of interest.
