# Supplementary material for: Agreement With Conjoined NPs Reflects Language Experience
Source: Front Psychol. 2018 Apr 19;9:489. doi: 10.3389/fpsyg.2018.00489 (PMC5917690; doi:10.3389/fpsyg.2018.00489)
Supplement: Supplementary file 1 [file Table_1.docx]

**Supplementary Materials.**

**Experimental preambles for Oral Production Task (Experiment 1)**

| **Count** | **Mass** |
| --- | --- |
| the authorization and examination | the assessment and management |
| the city and zipcode | the beauty and emotion |
| the conflict and de-escalation | the bicycling and running |
| the corporation and job | the bread and water |
| the cup and bowl | the cheese and milk |
| the deception and hijacking | the collaboration and cooperation |
| the discussion and settlement | the commercialization and globalization |
| the etching and carving | the copper and iron |
| the front and back | the corruption and extortion |
| the greeting and recognition | the desperation and unhappiness |
| the hardcopy and disk | the dust and mold |
| the interruption and intrusion | the equipment and machinery |
| the item and story | the exaggeration and lying |
| the lid and can | the food and agriculture |
| the middle and tip | the insulation and padding |
| the misunderstanding and reconciliation | the involvement and leadership |
| the name and address | the licensing and education |
| the operation and recovery | the loyalty and honor |
| the page and script | the mud and dirt |
| the reference and tutorial | the opposition and fighting |
| the rejection and disqualification | the painting and remodeling |
| the resignation and ruling | the power and success |
| the reversal and retraction | the preparation and cooking |
| the scolding and citation | the principal and interest |
| the start and end | the prose and poetry |
| the storyline and title | the rain and wind |
| the substitution and disruption | the rehabilitation and assistance |
| the summary and program | the shipping and handling |
| the syndrome and symptom | the silence and solitude |
| the username and password | the software and hardware |
| the viola and violin | the tea and coffee |
| the wedding and celebration | the weight and size |
| **Collectives** | **Collectives, continued…** |
| the album and soundtrack | the exhibit and gallery |
| the appendix and glossary | the galaxy and constellation |
| the arsenal and battlefield | the garden and orchard |
| the bookstore and café | the harbor and marina |
| the church and museum | the neighborhood and precinct |
| the collage and scrapbook | the playground and arcade |
| the directory and catalog | the prison and jail |
| the encyclopedia and thesaurus | the university and library |
