# Supplementary material for: Agreement With Conjoined NPs Reflects Language Experience
Source: Front Psychol. 2018 Apr 19;9:489. doi: 10.3389/fpsyg.2018.00489 (PMC5917690; doi:10.3389/fpsyg.2018.00489)
Supplement: Supplementary file 2 [file Table_2.docx]

**Supplementary Materials.**

**Stimuli for written production task (Experiment 2a and 2b)**

**Baseline task:**

Conjoined NPs

| the assistance and rehabilitation of the veteran |  |
| --- | --- |
| the corruption and extortion by the politician |  |
| the unhappiness and desperation of the losing team |  |
| the opposition and fighting in the senate |  |
| the copper and iron for the plumbing |  |
| the bicycling and running around the convention center |  |
| the etching and carving of the ancient tree |  |
| the insulation and padding from the cold weather |  |

**Story: (F = filler, P = prime, CB = counterbalance)**

F: On Thursday afternoon, representatives from companies all over the world began showing up at the convention center for the trade show, which featured new technology for construction.

P: The hardware and software for automated design (was/were/would be) the main attraction. CB: The prototype robot/s (was/were being/had been) shipped in from Tokyo for its/their unveiling.

F: When the guests arrived for the trade show, they would be hungry.

P: For a large event like this, the preparation and cooking (takes/take/took) hours,

CB: and the chef/chefs (was/were cooking/cooked) all morning to get everything ready for the guests.

P: The tea and coffee (was/were/had been) spread out on a long table, along with some muffins and cookies,

CB: but the ice cream/ice cream sundaes (was still/were still/stayed) in the freezer.

F: The maintenance staff had been working on finishing renovations.

P: In the lobby, the painting and remodeling (was/were taking/had taken) a long time.

CB: However, the exhibition hall/s was/were/looked ready to go.

F: After the contractors left,

P: the mud and dirt from their job (was/were/had been) removed by cleaning crews.

CB: Overall, the interior designer/s (was/were/felt) very happy with the results.

F: In the atrium, cleaning still needed to be done,

P: as the dust and mold (was/were/had been) getting out of control.

CB: The cleaning supplies/equipment (was/were being/had been) quickly dispatched to make everything spotless.

F: In the next block over, a publishing company had been working on a program for the convention.

P: The education and licensing of every contractor (was/were/had been) listed in the program, CB: and the editor/editors (was/were making/made) sure that no mistakes had been made.

F: The publishing company also needed to put together a handbook for the students volunteering at the convention.

P: The tutorial and reference for the new technology (was/were/had been) bundled with the handbook.

CB: The students/volunteer staff (was/were/had been) enlisted to serve as guides for the attendees of the convention.

**Story Completion Task: (F = filler, P = prime, CB = counterbalance, T = Target)**

1) CB: The convention/talks (was/were kicked off/kicked off) with a speech from a major CEO of …

P: The recognition and appreciation of the planning committee (was/were proceeding/proceeded) …

F: The CEO discussed changes to the organization’s by-laws, which happened because…

T: Everyone felt nervous, because the retraction and reversal of the motion…

2) CB: Other speakers/another speaker (was/were talking / talked) about characteristics that contributed to…

P: The honesty and reliability of a company (was/were/proved to be) very …

F: The audience generated new ideas for …

T: The speakers praised the attendees, because the collaboration and cooperation in the audience…

3) CB: Out in the exhibition hall, people/someone (was/were/had been) shouting over …

P: The speed and capacity of the new prototype (was/were being/had been) questioned by…

F: During the demonstration, the new machine did not lift…

T: The exaggeration and lying by the company…

4) CB: Another booth/other booths (was/were recruiting/recruited) people to run for office in…

P: The purpose and mission of the trade organization (was/were/had been) clearly defined in…

F: Recruiters talked about the many incentives for becoming an officer, including…

T: The involvement and leadership of the executive committee…

5) CB: At the trade show, a bank / several banks (was/were advertising / advertised)…

P: The qualification and approval for a loan (was/were/could be) done…

F: A loan calculator helped everyone figure out…

T: The principle and interest for the loan…

6) CB: Once they had their credit information, the crowd / shoppers (was/were able to make/could make) their purchases of…

P: The weight and size of a machine (was/were/had been) used to determine…

F: Potential buyers spoke with…

T: The discussion and settlement around the cost structure…

7) CB: During the convention, many orders/an order (was/were/had been) placed for …

P: The set-up and configuration for a robot (was/were/had been made) available …

F: A representative from the company handled the delivery information, collecting …

T: The shipping and handling of the robot…

8) CB: At the end of the day, the cleaning crew/the cleaners (was/were in a rush/rushed) to …

P: The supervision and management of the cleanup procedure (was/were/had been) handled by …

F: Vendors needed to load all of their products on…

T: The equipment and machinery from the exhibition…
